# Supplementary material for: Concretized structural evolution supported assembly-controlled film-forming kinetics in slot-die coated organic photovoltaics
Source: Nat Commun. 2023 Oct 9;14:6312. doi: 10.1038/s41467-023-42018-7 (PMC10562442; doi:10.1038/s41467-023-42018-7)
Supplement: Supplementary file 1 — Supplementary Information [file 41467_2023_42018_MOESM1_ESM.pdf]

Supplementary information to

Concretized Structural Evolution Supported Assembly-Controlled Film-Forming Kinetics in Slot-die Coated Organic Photovoltaics

Contents

Supplementary Table and Figures 2

Supplementary note 1 – Absorption Edge Fitting of In-situ UV-vis Spectroscopy 15

Supplementary note 2 – Fitting of GIWAXS peaks. 18

Supplementary note 3 – Molecular Dynamic Simulation Analysis of Y6, N3, and L8-BO 20

Supplementary note 4 - Discussion on effect of temperature, donor, and solvent concentration to film forming kinetics 23

Supplementary note 5 – Film forming kinetics in Toluene 26

References 28

Supplementary Tables

Supplementary Table 1 Performance of 1 cm^2 OPV devices

Donor	Temperature (°C)	Acceptor	V_{oc} (V)	J_{sc} (mA/cm^2)	FF (%)	PCE (%)
PM6	40	Y6	**0.743**±0.009 (0.756)	**8.42**±0.33 (8.48)	**48.89**±3.20 (52.29)	**3.06**±0.28 (3.35)
		N3	**0.667**±0.077 (0.662)	**1.33**±0.28 (1.95)	**26.09**±1.08 (26.67)	**0.233**±0.060 (0.344)
		L8-BO	**0.810**±0.009 (0.812)	**20.65**±1.02 (21.58)	**59.71**±4.14 (64.94)	**10.00**±0.98 (11.37)
	60	Y6	**0.741**±0.009 (0.747)	**11.21**±0.31 (11.5)	**51.97**±2.56 (54.5)	**4.32**±0.33 (4.68)
		N3	**0.687**±0.043 (0.738)	**1.34**±0.14 (1.62)	**25.22**±1.71 (28.94)	**0.234**±0.048 (0.346)
		L8-BO	**0.804**±0.008 (0.81)	**20.46**±0.61 (20.68)	**64.56**±5.24 (69.99)	**10.61**±0.88 (11.72)
	80	Y6	**0.733**±0.013 (0.755)	**15.74**±1.29 (17.61)	**52.74**±5.54 (60.63)	**6.09**±0.89 (8.05)
		N3	**0.640**±0.079 (0.776)	**2.57**±0.84 (4.2)	**26.07**±2.29 (29.3)	**0.459**±0.244 (0.954)
		L8-BO	**0.799**±0.004 (0.802)	**20.68**±0.84 (21.78)	**66.94**±1.71 (67.68)	**11.06**±0.52 (11.81)
	100	Y6	**0.726**±0.012 (0.743)	**18.14**±1.09 (19.46)	**57.44**±3.04 (60.06)	**7.57**±0.68 (8.69)
		N3	**0.598**±0.186 (0.755)	**6.73**±2.28 (9.82)	**30.79**±4.23 (32.56)	**1.29**±0.67 (2.41)
		L8-BO	**0.807**±0.004 (0.803)	**21.28**±0.84 (21.97)	**67.18**±1.80 (68.97)	**11.53**±0.36 (12.17)

1. Values in bracket are the parameters of device with highest PCE.

2. Average values are calculated from at least 5 independent 1 cm^2 devices.

Supplementary Table 2. Performance of 0.04 cm^2 OPV devices spin-coated with *o*-xylene.

Donor	Annealing	Rotation Speed (Rpm)	Acceptor	Voc (V)	Jsc (mA/cm^2)	FF (%)	PCE (%)
PM6	110 °C 10min	3000	Y6	**0.769**±0.001<br>**0.770**	**17.536**±0.142<br>**17.745**	**65.961**±0.803<br>**66.436**	**9.544**±0.160<br>**9.738**
			N3	**0.800**±0.001<br>**0.800**	**20.171**±0.151<br>**20.021**	**68.980**±1.826<br>**67.154**	**11.934**±0.398<br>**11.536**
			L8-BO	**0.845**±0.007<br>**0.850**	**21.487**±0.391<br>**21.863**	**74.260**±0.406<br>**74.605**	**14.464**±0.453<br>**14.870**
		3500	Y6	**0.767**±0.002<br>**0.769**	**16.826**±0.237<br>**17.179**	**66.339**±0.629<br>**67.222**	**9.186**±0.222<br>**9.528**
			N3	**0.802**±0.002<br>**0.803**	**20.035**±0.304<br>**20.275**	**74.481**±0.373<br>**74.412**	**12.832**±0.169<br>**12.997**
			L8-BO	**0.848**±0.003<br>**0.847**	**22.412**±0.392<br>**22.675**	**75.508**±0.474<br>**75.964**	**15.396**±0.269<br>**15.647**
		4000	Y6	**0.766**±0.004<br>**0.769**	**16.207**±0.400<br>**16.689**	**66.187**±0.845<br>**66.872**	**8.807**±0.223<br>**9.203**
			N3	**0.800**±0.003<br>**0.799**	**19.337**±0.500<br>**19.453**	**71.655**±1.588<br>**73.044**	**11.889**±0.498<br>**12.181**
			L8-BO	**0.849**±0.003<br>**0.851**	**22.228**±0.272<br>**22.619**	**75.204**±1.353<br>**76.938**	**15,230**±0.455<br>**15.878**
	w/o.	3000	Y6	**0.772**±0.005<br>**0.777**	**16.777**±0.174<br>**16.887**	**64.546**±0.350<br>**65.133**	**8.962**±0.182<br>**9.171**
			N3	**0.826**±0.002<br>**0.827**	**19.227**±0.210<br>**19.294**	**71.869**±1.296<br>**73.062**	**12.240**±0.325<br>**12.506**
			L8-BO	**0.861**±0.004<br>**0.868**	**20.492**±0.119<br>**20.299**	**74.134**±0.266<br>**74.548**	**14.020**±0.063<br>**14.080**
		3500	Y6	**0.776**±0.001<br>**0.777**	**16.292**±0.373<br>**16.595**	**65.049**±0.674<br>**65.787**	**8.822**±0.224<br>**9.092**
			N3	**0.821**±0.002<br>**0.820**	**19.367**±0.215<br>**19.698**	**71.072**±1.759<br>**71.625**	**12.119**±0.347<br>**12.414**
			L8-BO	**0.860**±0.002<br>**0.862**	**21.382**±0.216<br>**21.606**	**74.333**±0.485<br>**73.995**	**14.659**±0.154<br>**14.773**
		4000	Y6	**0.771**±0.006<br>**0.773**	**15.273**±0.237<br>**15.613**	**63.580**±3.904<br>**66.934**	**8.040**±0.649<br>**8.664**
			N3	**0.817**±0.004<br>**0.821**	**17.294**±0.632<br>**18.120**	**70.848**±1.548<br>**72.641**	**10.747**±0.598<br>**11.589**
			L8-BO	**0.866**±0.002<br>**0.869**	**22.110**±0.036<br>**22.126**	**75.394**±0.205<br>**75.653**	**15.490**±0.080<br>**15.600**

1. Values on the second line are the parameters of device with highest PCE.
2. Average values are calculated from at least 4 independent 0.04 cm^2 devices.

Supplementary Figures

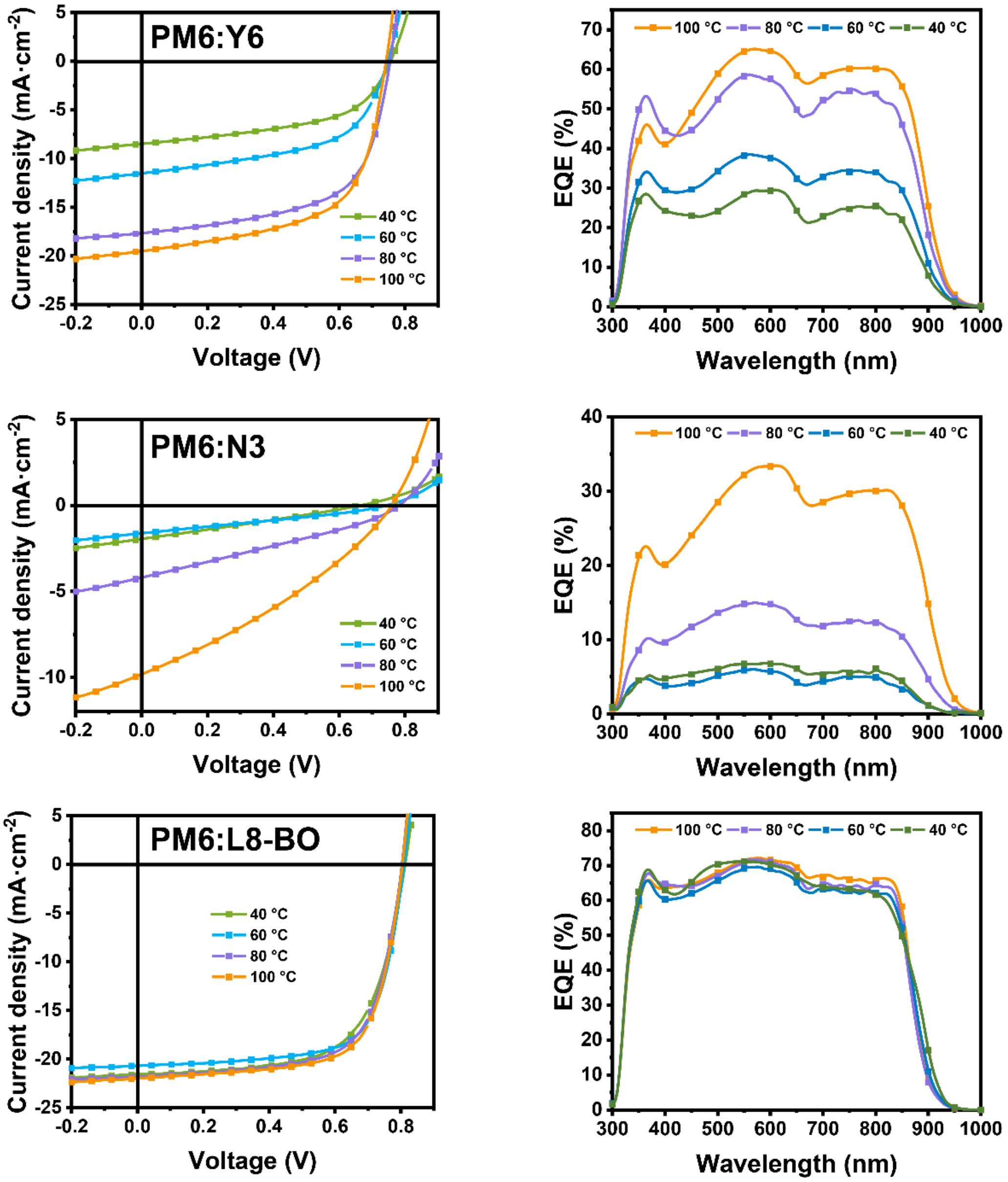

Supplementary Figure 1 EQE and JV curve for best cells coated under each condition.

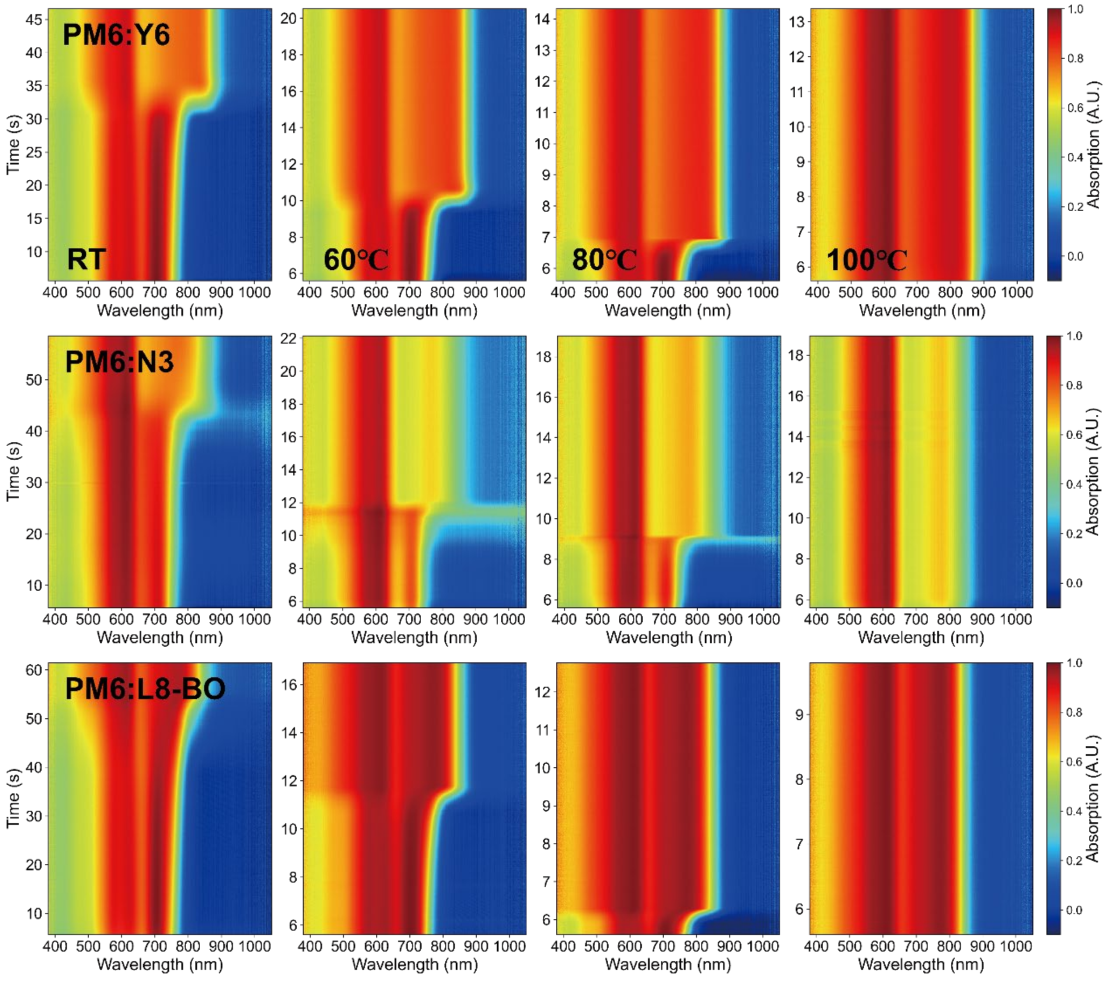

Supplementary Figure 2 In-situ UV-vis spectrum of blends coated under various temperature.

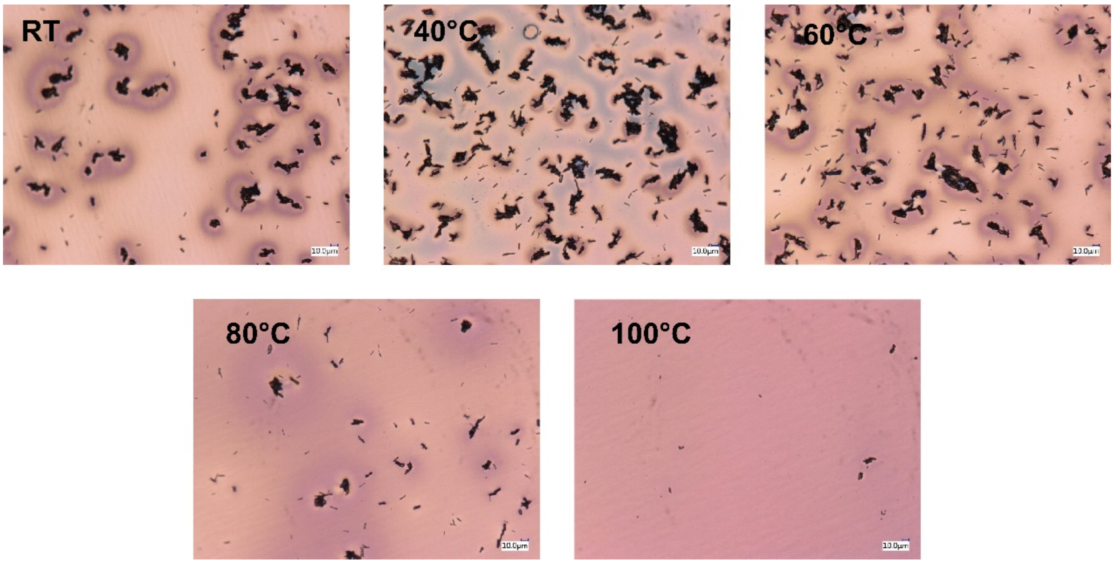

Supplementary Figure 3 Optical microscope image of PM6:N3 blend film coated

under various substrate temperature.

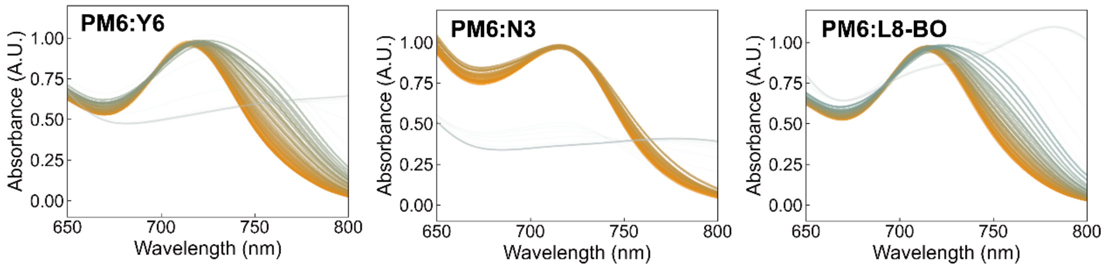

Supplementary Figure 4 Normalized in-situ UV-vis spectrum of the blends in stage one.

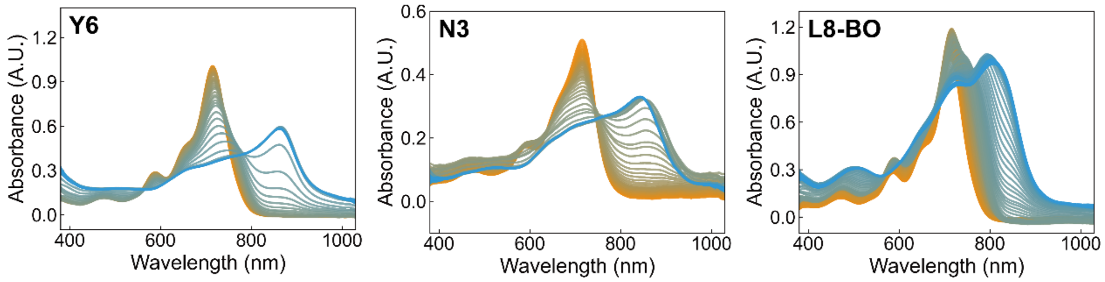

Supplementary Figure 5 In-situ UV-vis spectrum of pristine acceptor films coated at 40℃.

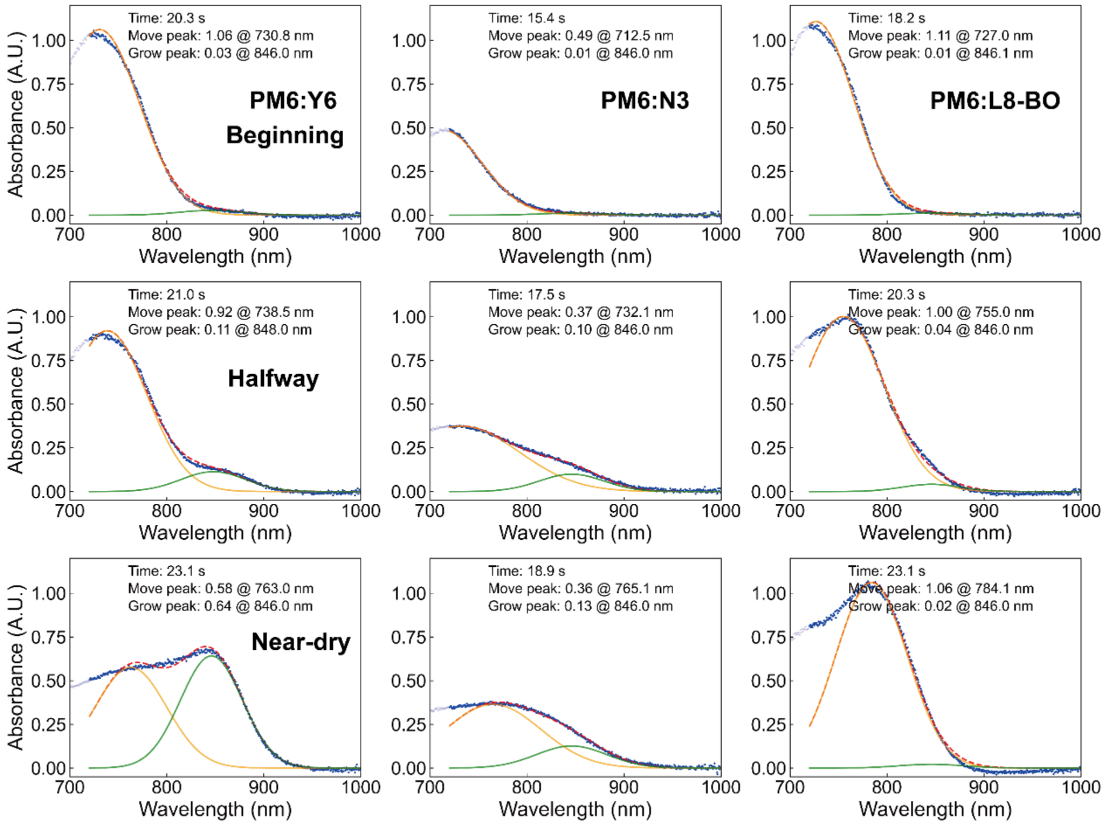

Supplementary Figure 6 Snapshots of in-situ UV-vis spectrum of blends showing beginning phase, halfway, and near-dry phase of solvent evaporation. Orange line indicates the position of moving peak while green line indicates the growing peak.

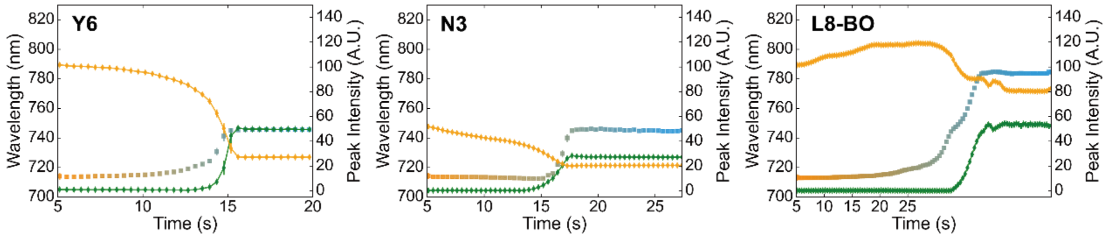

Supplementary Figure 7 Fitting result of in-situ UV-vis spectrum of pristine

acceptor film coated at 40°C.

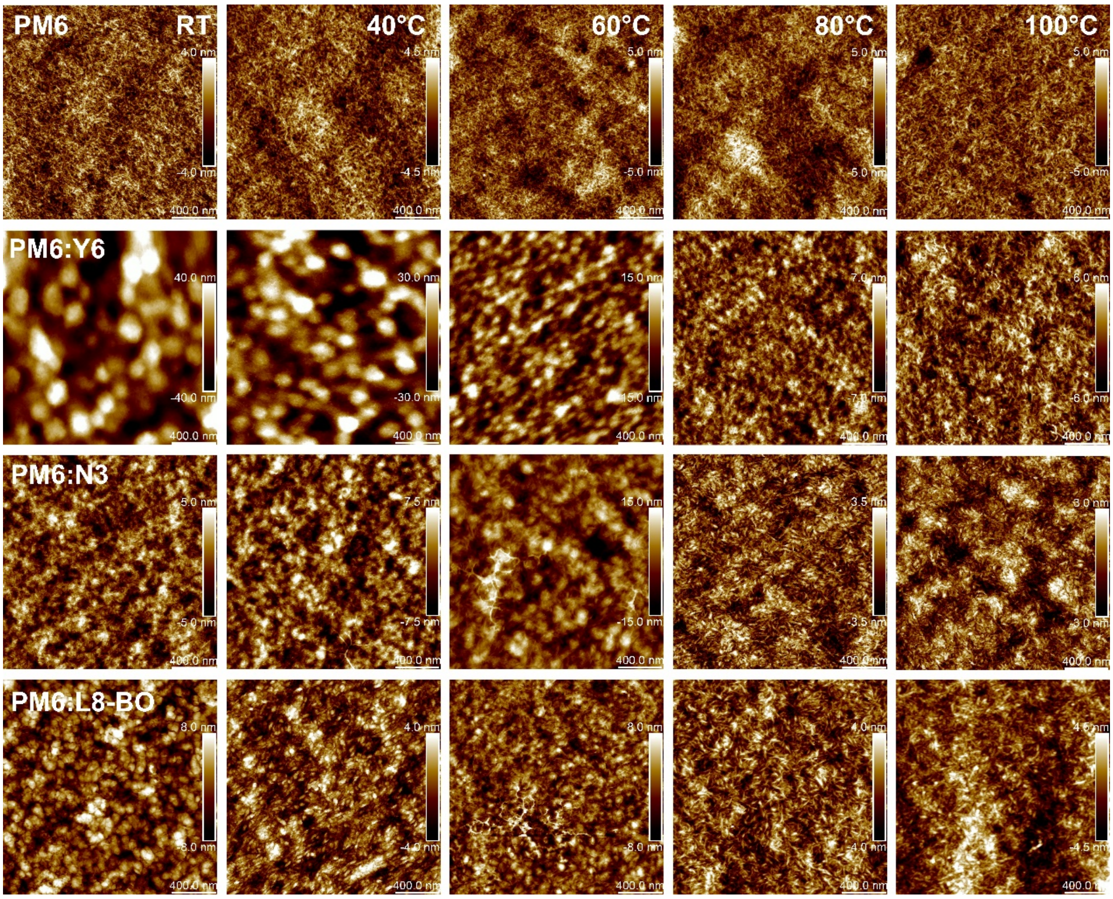

Supplementary Figure 8 AFM images of PM6 film and blend films coated under various temperature.

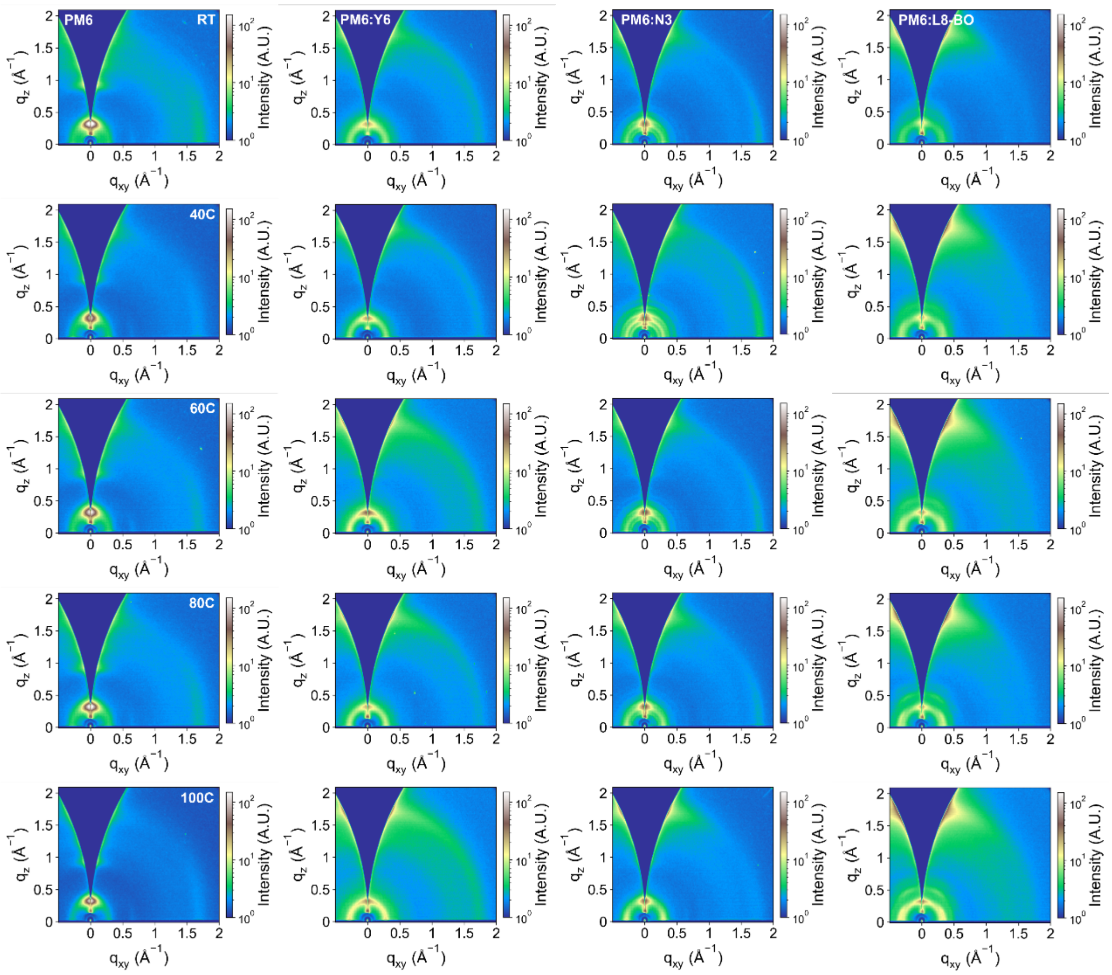

Supplementary Figure 9 GIWAXS images of pristine PM6 films and blend films coated under various temperatures.

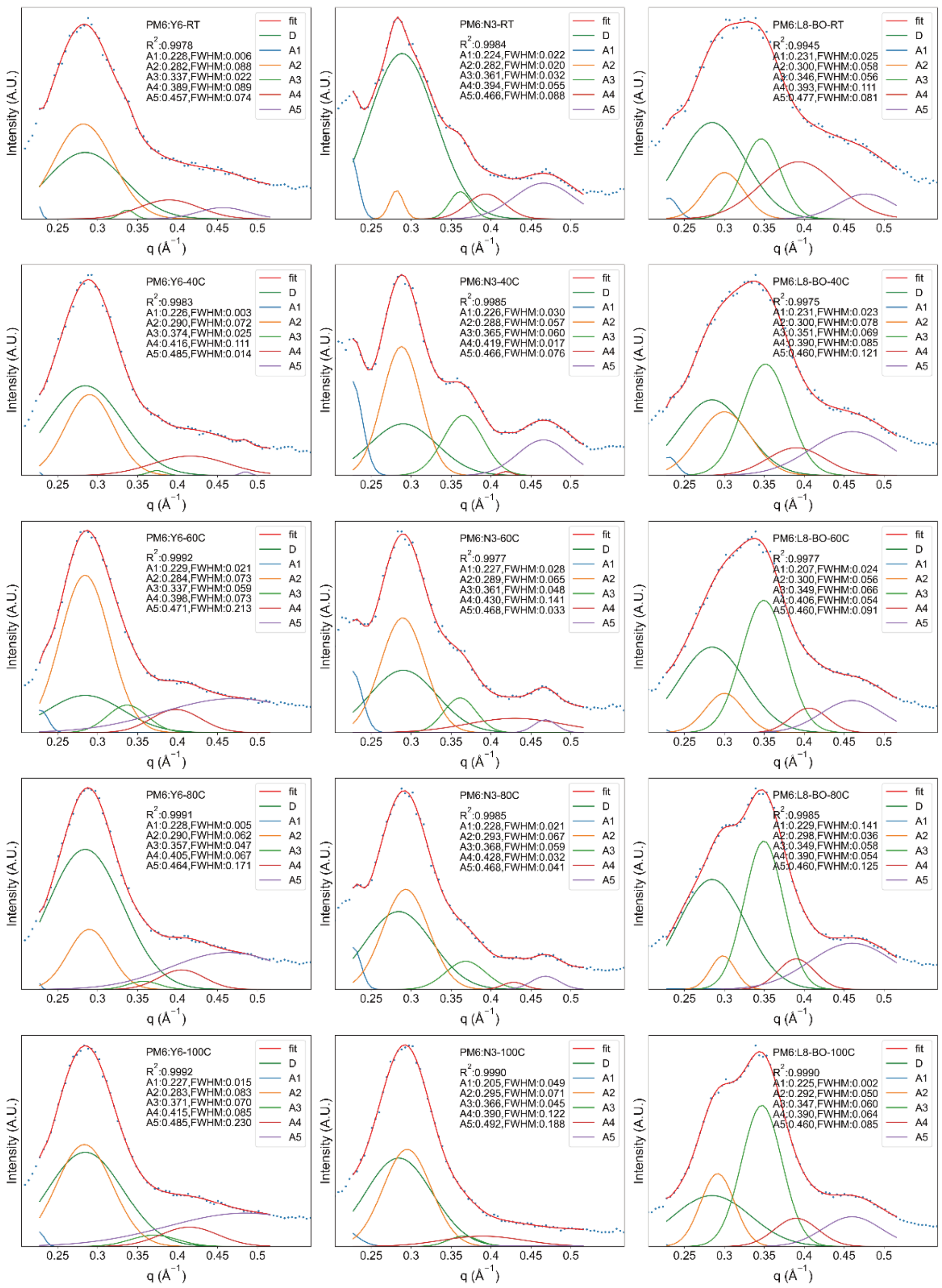

Supplementary Figure 10 GIWAXS in-plane fitting results of blend films coated under various temperatures.

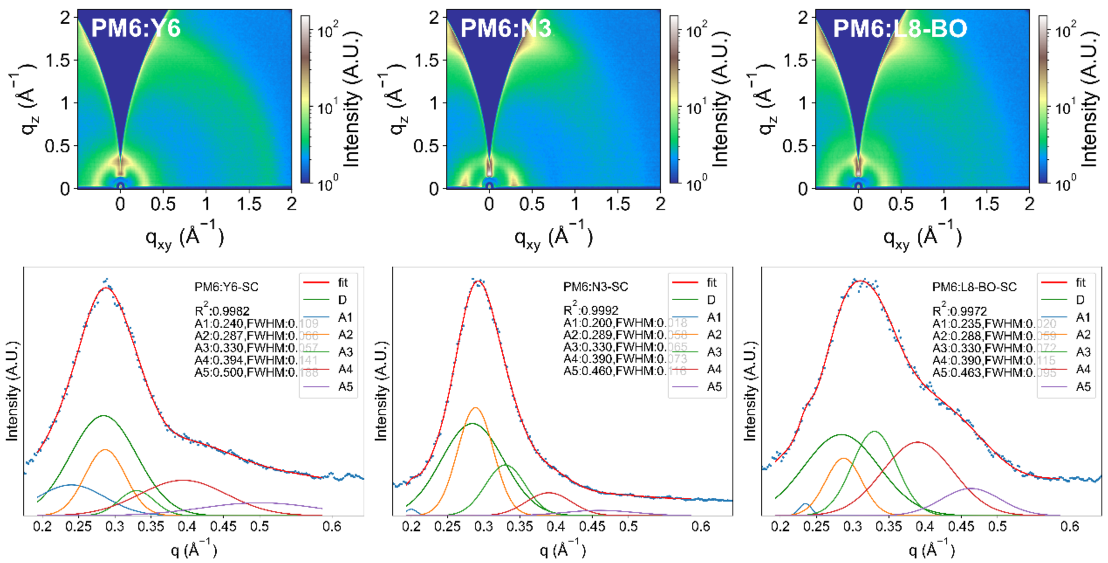

Supplementary Figure 11 GIWAXS in-plane fitting results of blend films spin-coated with o-xylene.

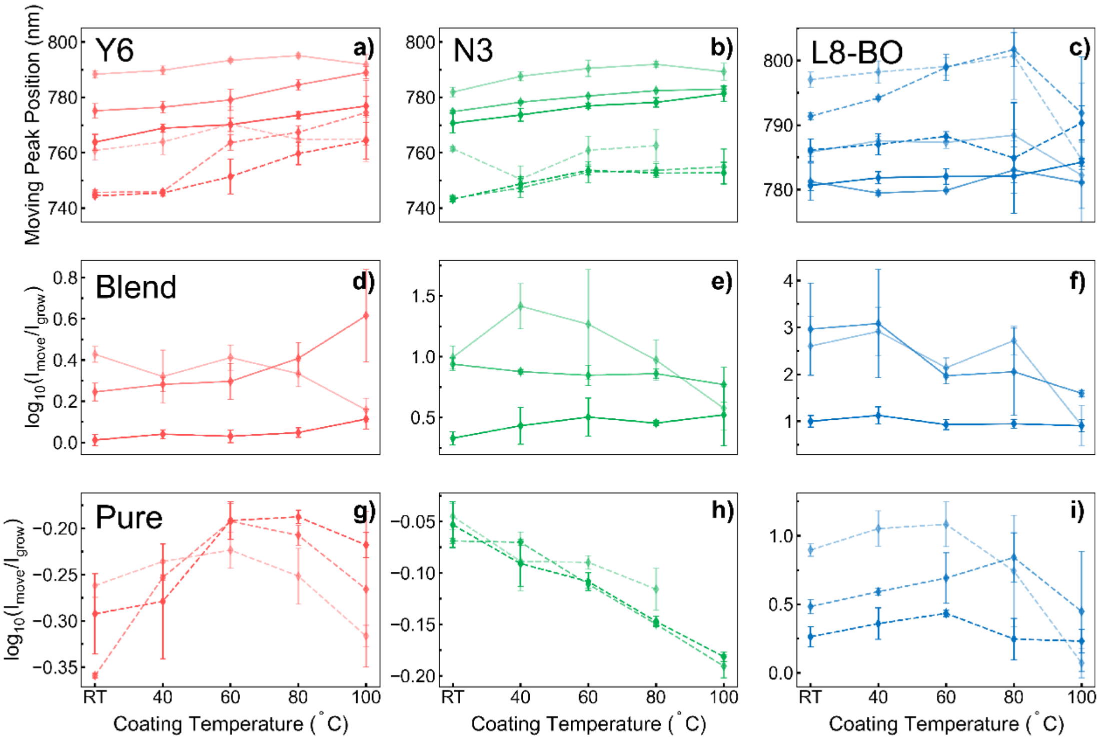

Supplementary Figure 12 Statistical analysis of fitted in-situ UV-vis spectrums. **(a-c)** position of moving peak in dry film of different systems. Solid lines are moving peaks fitted from blends while dashed lines are from pristine films. Thinner color represents solutions of lower concentration. **(d-f)** Comparison of intensity between

growing peak and moving peak in blend films. **(g-i)** Comparison of growing peak and moving peak in pristine films. Every datapoints were statistically derived from at least 3 independent experiments.

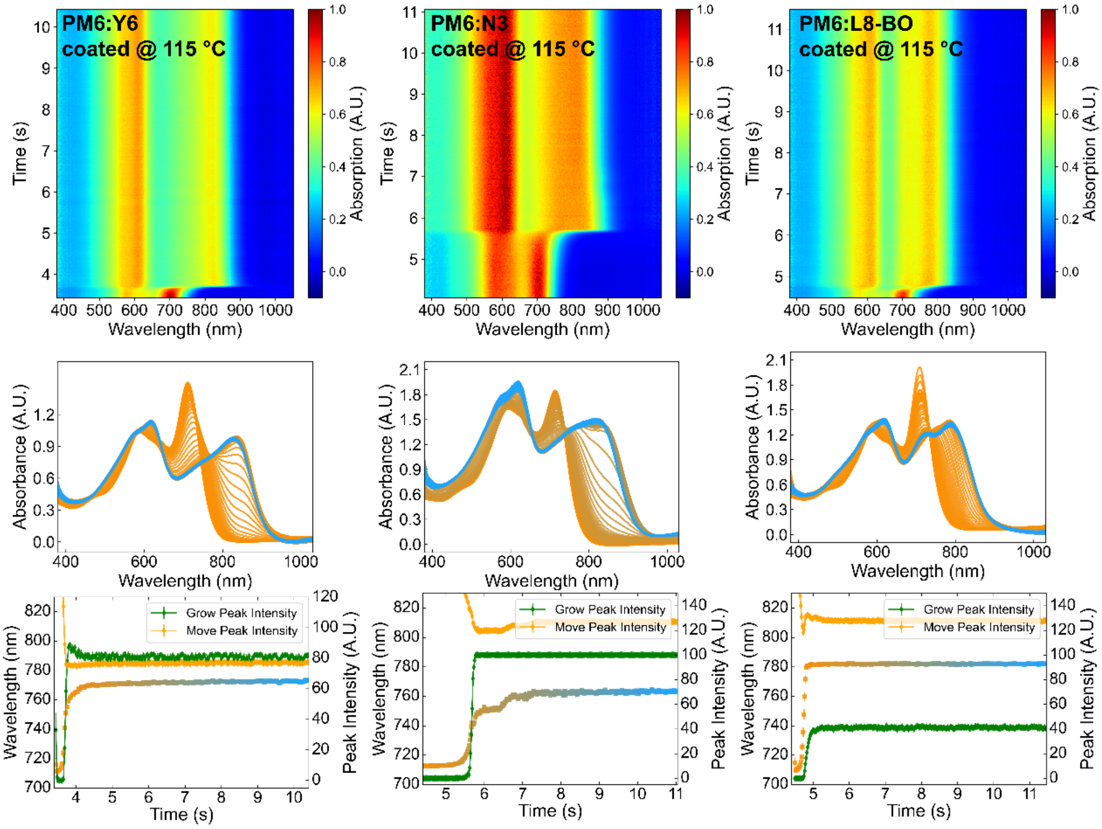

Supplementary Figure 13 Film forming kinetics of organic blend film coated on glass substrate at 115°C.

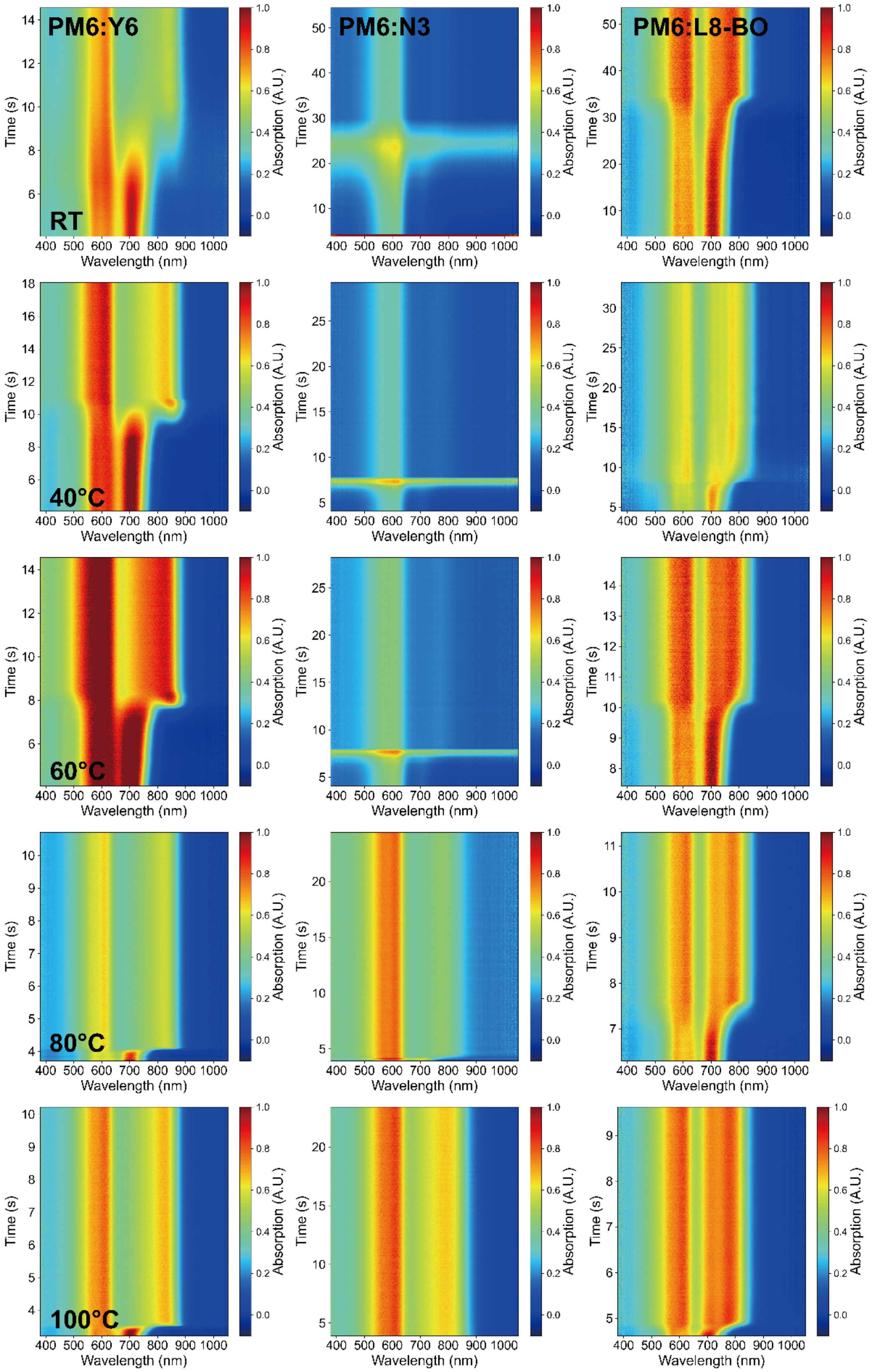

Supplementary Figure 14 In-situ UV-vis spectrum of blends coated with toluene under various temperature.

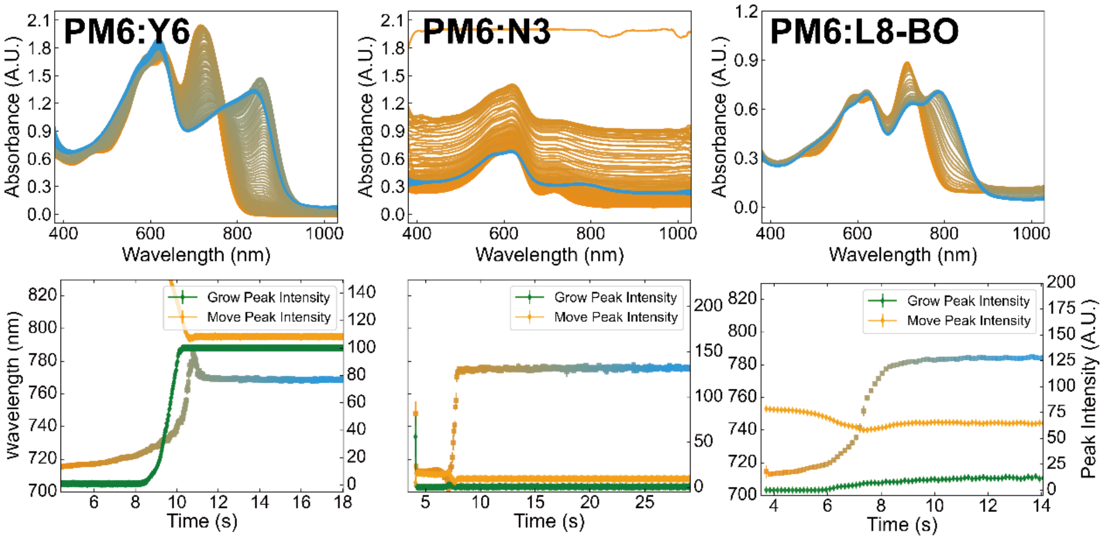

Supplementary Figure 15 Stack plot and fitted spectrum of blends coated with toluene under 40 °C.

Supplementary note 1 – Absorption Edge Fitting of In-situ UV-vis Spectroscopy

Each line series of time resolved UV-vis spectroscopy of a slot-die coating session is transformed into absorbance (as shown in fig.2d-f) for peak fitting according to following equation:

$$A(\lambda, t) = -\ln\frac{T(\lambda, t)}{T_0(\lambda)} \tag{1}$$

Where $T(\lambda, t)$ is spectrometer recorded intensity at given wavelength and time and $T_0(\lambda)$ is baseline intensity of the light source.

Our area of interest is restricted in absorption edge across the whole film forming process. Thus, a spectrum region starting from λ_{0-0} peak of acceptor to 1000 nm is designated. Since the distinguish of Y6's λ_{0-0} peak in solution and film, we limited the lower boundary to 700 nm.

Two gaussian peaks are used to fit the absorbance spectrum within the defined region above: One movable peak and a fixed peak. The initial value and fitting boundaries are given in **Supplementary Table 3**.

**Peak**	**Movable Peak**			**Fixed Peak**		
**Item**	**Position (nm)**	**Intensity (A.U.)**	**Sigma**	**Position (nm)**	**Intensity (A.U.)**	**Sigma**
**Initial Guess at t = 0**	λ_{0-0}	$A(\lambda_{0-0})$	10	λ_{fixed}	$A(\lambda_{fixed})$	33
**Initial Guess at t**	λ_{t-1}	A_{t-1}	σ_{t-1}	λ_{t-1}	A_{t-1}	σ_{t-1}
**Boundary**	700, 820	0, 2	σ_{t-1}-5, σ_{t-1}+5	λ_{fixed}-1, λ_{fixed}+1	A_{t-1}-0.08, 1	23, 43

Supplementary Table 3 fitting parameters and boundaries of two peaks.

All spectrums were fitted starting from ~5.6s after start of the coating session and till the end of acquisition. The first curve was fitted using initial guess at t = 0, while rest of the curves were fitted using result of last fit as initial guess, according to time series. For each curve, a mixed error function using both Root Mean Square Error and R-square error was applied, and Sequential Least Squares Programming (SLSQP) method was used to minimize the fitting loss. The whole fitting procedure is conducted with python using scipy[1] and numpy[2] package.

The restriction on lower bound of growing peak is set allowing instrumental error and subtle decrease on peak strength, or numerical instability will occur between moving peak sigma and grow peak strength, which is unphysical for a consecutive process to happen.

Though an obvious growing peak can be distinguished in Y6 films, it is rather hard to directly determine the position of grow peak for N3 & L8-BO based films. Thus, we scanned the grow peak position from 830 nm to 880 nm to find a proper position. The result of the scan is shown in **Supplementary Figure 16**. It turned out that a grow peak position at ~847 nm possesses some sort of special trait, which is the transition point of loss curve for both N3 & L8-BO based film forming process, and also around the lowest loss peak position for Y6 based film forming process. We thus set λ_{fixed} at 847 nm in our further analysis.

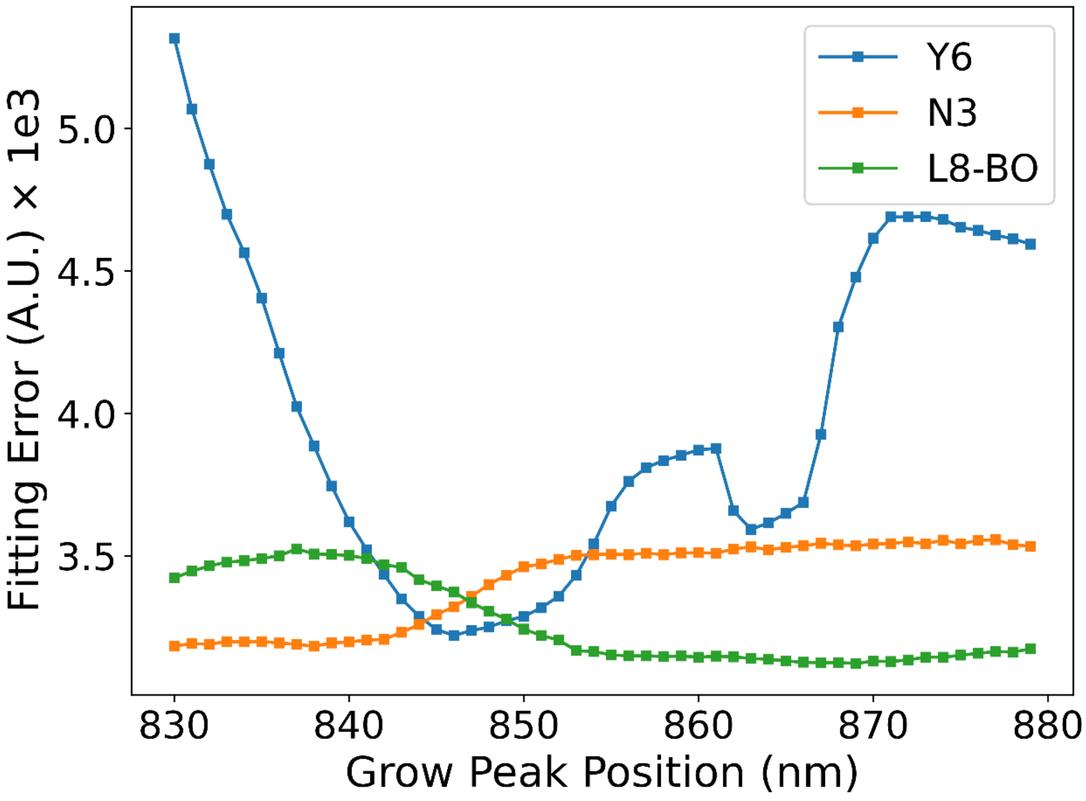

Supplementary Figure 16 Fitting error of peak scanning.

Supplementary note 2 – Fitting of GIWAXS peaks.

We took GIWAXS images for pristine PM6 film and blend films coated from room temperature to 100°C and results are shown in **Supplementary Figure 9**.

The fitting of GIWAXS patterns starts by fitting peaks of pristine PM6 film coated under same temperature as blend films. After determining the peak position and FWHM of PM6 film, the results are then used in fitting of PM6:N3 film. We choose the fitting result of PM6:N3 film as initial guess of PM6:Y6 and PM6:L8-BO's film since PM6:N3 film shows the most varied acceptor peaks in three Y6 analogues studied.

All IP direction fittings we fit the GIWAXS data from 0.228 $Å^{-1}$ to 0.520 $Å^{-1}$, a total of 63 data points in our experimental setup. While OOP direction we fit 1.100 $Å^{-1}$ to 2.144 $Å^{-1}$ with a total of 225 data points. Gaussian peak was chosen to model the scattering signal. Only RMSE is minimized during the fitting procedure and SLSQP method was used to minimize the fitting loss.

We first tried to fit PM6:N3 film coated at room temperature, results of the fitting are shown in **Supplementary Table 4**.

Supplementary Table 4 Fitting result of PM6:N3 film coated at room temperature.

	**Donor OOP-1**	**Donor OOP-1**	**Donor IP**	**N3-IP-1**	**N3-IP-2**	**N3-IP-3**	**N3-IP-4**	**N3-IP-5**
**Peak Position ($Å^{-1}$)**	1.2071	1.6714	0.2845	0.2257	0.2877	0.3662	0.4083	0.4662
**FWHM ($Å^{-1}$)**	0.4078	0.3632	0.1144	0.0302	0.0547	0.0562	0.0365	0.1086

Further fittings were conducted by applying the result of acceptor peaks in

Supplementary Table 4 and corresponding PM6 peaks as initial guess. Because of the uncertainty in peak strength and overlapping position of PM6-IP peak and N3-IP-2 peak, fitting result of these two peaks suffers numerical instability in strength. But non-overlapping peaks all fitted well, results are shown in **Supplementary Figure 10**.

Through fitting of various blend films coated under different temperature, it is worth noting that acceptor peaks we chosen from PM6:N3 blend coated under room temperature could matches with all other films.

The quantitative analysis of relationship between GIWAXS scattering pattern and coating temperature is not available due to the un normalized thickness of films, but qualitatively speaking, general similarity exists between scattering patterns of same PM6/Y6 analogue blend films. We thus focused on detailed explaining of GIWAXS pattern of films coated at 40°C.

The same analysis scheme is also applied to spin coated GIWAXS samples of blend film, result of which is shown in **Supplementary Figure 11**.

Supplementary note 3 – Molecular Dynamic Simulation Analysis of Y6, N3, and L8-BO

DFT calculation of molecules were done with Gaussian 09D package at at ωB97XD/6-31 G(d,p) level[3].

The statistic of dimer types was done with the assist of Machine Learning (ML) techniques. We first analyzed two frames from Y6's trajectory, gathering a total of 1476 bimolecular configurations and classified them into 10 types of dimers according to our previous report[4, 5]. **Supplementary Figure 17** showed examples of these dimers and a distribution statistic from the dataset.

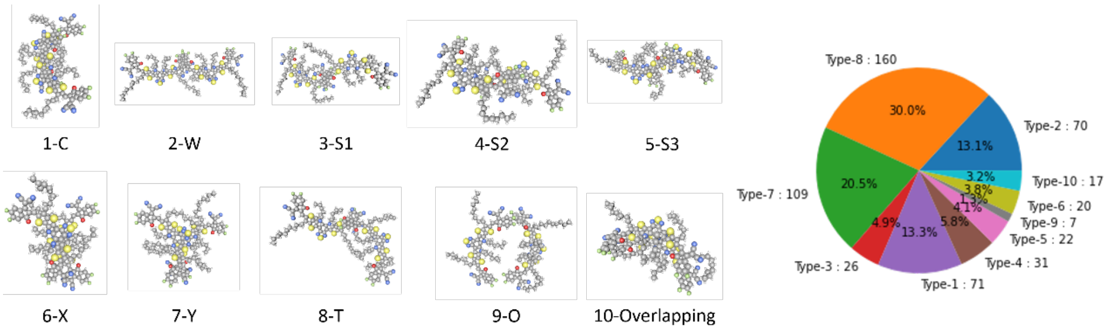

Supplementary Figure 17 Example of interacting dimers and statistic distribution in dataset

After manually generating the dataset need to train the ML model, feature vector of packing dimers was extracted including: 1. Position vector between two molecule's mass centers (black vector of **Supplementary Figure 18**). 2.cosine value between principal axis of two molecules. 3. Shortest position vector between A-groups of two molecule (green vector). 4. Shortest position vector between A-group of a molecule and

A'-group of another (blue vector).

Calculation of the principal axis of an acceptor molecule follows these steps:

1. Determine the geometrical center for terminal groups of an acceptor molecule. In our case, terminal group refers to 2-(5,6-difluoro-2-methylene-3-oxo-2,3-dihydro-1H-inden-1-ylidene)malononitrile units of Y6 analogues.
2. Set the line determined by the two geometrical centers as X-axis of the principal axis, and the midpoint of the line as principal center.
3. Determine Y axis. The Y axis would be the line in such a plane that: I. contains both geometrical centers, II. the summation of distance those backbone atoms to the plane reaches the minimum. Then, the Y axis will be the perpendicular line that intersect X axis at principal center.
4. Calculate Z axis. The Z axis is finally determined by:

$$z = x \times y \tag{2}$$

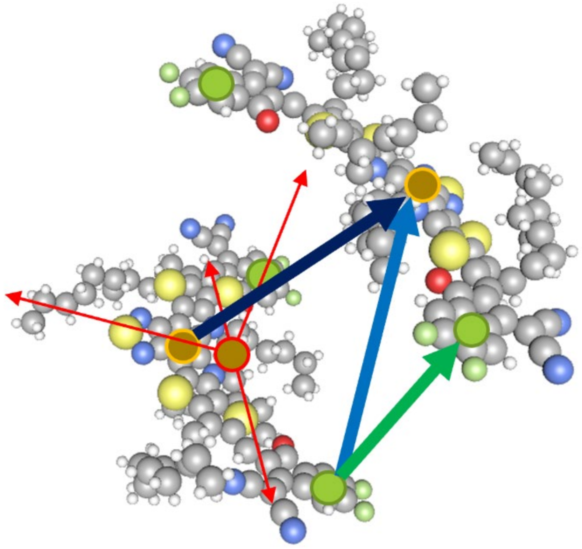

Supplementary Figure 18 Sketch of feature vectors between two molecules.

After generating the feature vector, a Random Forest Classifier with 100 independent

estimators was trained. The minimum sample per leaf was set to 2. ML model was used from scikit-learn package[6] without any modifications. After training, 10-fold cross validation of 50 times indicates an accuracy of 83% ± 4%. The ML model was then used to automatically analyze each frame of the MD trajectory, extracting every valid dimer pairs. Finally, the ten types of dimers were categorized into A-X type dimer, A-D,A' dimer, and A'-A' dimer, based on interacting subgroup of the pair.

The gathered dataset and code used to generate feature vector can be found in code availability section of the report.

Supplementary note 4 - Discussion on effect of temperature, donor, and solvent concentration to film forming kinetics

Now we discuss the influence of external conditions of coating procedure on film forming kinetics. In **Supplementary Figure 12** we plot moving peak destination and Intensity ratio of moving peak and growing peak for films coated under various conditions. We coated pure acceptor films with a concentration gradient of 8.73 mg/mL, 4 mg/mL, and 1 mg/mL, while blend film with D: A=1:1.2, 16 mg/mL (8.73 mg/mL acceptor), D: A=3:1 16 mg/mL (4 mg/mL acceptor), and D: A=3:1, 4 mg/mL (1 mg/mL acceptor) for blend films. Blends are represented in solid lines and pure acceptor films are represented in dashed lines. Color gradient is correspondent to concentration gradient.

We first discuss the effect of donor on acceptor evolution. For all three blends an increment in $\log(I_{move}/I_{grow})$ value was observed in all conditions. In Y6 and N3's case, introduction of donor brings such value from negative to positive, which means the major evolution mechanism shifted from growing to moving. In L8-BO's case, introduction of donor further enhanced the trend of moving, increasing its intensity to at least one magnitude higher than that of growing peaks as shown in **Supplementary Figure 12f**. Such shifting could be explained by intermolecular interaction between donor and acceptor molecules. An absorbing action, which acceptor molecule was absorbed onto polymer fibrils with its backbone stick to polymer surface, would severely influence the possibility of forming different dimers. In this scenario, vacancy

for dimer **2c** and **2d** to generate will be halved, while **2b** would be less affected. By comparing the result of two blend with 16 mg/mL concentration but different D/A ratio and their pure acceptor solution counterparts, further increment in the moving peak ratio is observed and such trend cannot be explained simply be acceptor concentration change. This further confirmed our conclusion that donor-acceptor interaction would influence the acceptor evolution. However, such interaction is not harmless to **3a** type micro aggregates. In **Supplementary Figure 12a** and **Supplementary Figure 12b** we saw that donor facilitated moving peak of Y6 and N3 move toward a redder position by around 20~30 nm in average, but inverted effect was observed for L8-BO in **Supplementary Figure 12c.** Introduction of donor polymer blue shifted L8-BO's moving peak around ~10nm, which could indicate breaks in long acceptor chains.

Concentration and coating temperature have synergic effect on film forming kinetics. In pure acceptor films, a maximum in $\log(I_{move}/I_{grow})$ can be found in Y6 and L8-BO coated under different coating temperature and as solvent concentration decreases, the temperature where max $\log(I_{move}/I_{grow})$ was achieved is lowered. For N3, a monotone decreasing is observed (**Supplementary Figure 12h**). In blends all three acceptors exhibited different behavior compared with pure film. For film coated with common device D/A ratio and concentration, the variation of $\log(I_{move}/I_{grow})$ is not as intense as in pure film, but the same fluctuation is observed for blend under lower concentration. We consider this synergic effect of concentration and coating temperature is an equilibrium between solvent evaporation and molecule diffusion. When films are coated under low temperature, slow solvent evaporation allows more

time for acceptors to reach a more thermodynamically favorable configuration, i.e., more condense packing motif. As temperature raises, reduced organization time and increased solvent thermos movement make acceptors could only chain up with each other in more **2b** type aggregation. If solvent evaporates too fast and there is no time for acceptors for organization, they will have to aggregate on-site and seldom have change to develop a chain structure. For a solution with lowered concentration, possibility of forming dimers are greatly reduced. But the formation of **3a** type micro aggregation is not as much affected compared with **3b** and **3c** type. Such shift in balance results in increment in portion of peak movement. If acceptors have no chance to organize, lower the concentration would result in lower chance of chaining up, which inversely increase the portion of growing mechanism observed in higher temperatures, compared with films coated with higher concentration. For pure N3 coated film in **Supplementary Figure 12h**, most of acceptors might have crystallized into large crystals and does not affect film forming kinetics in later stages. Thus, concentration in solution actually formed a film might be much lower, resulting in similar tendency that aggregates more as solvent evaporates faster. In such concentration, increment of solvent thermodynamic movement helps little in formation of 2a type dimer, since acceptors are too far from each other to form large quantity of dimers.

Supplementary note 5 – Film forming kinetics in Toluene

Film forming kinetics of PM6:Y6, PM6:N3, and PM6:L8-BO systems in Toluene was recorded using the in-situ UV-vis spectroscopy and analyzed with the proposed model as described previously. The waterfall plots of all spectrums were shown in **Supplementary Figure 14.** Compared with that coated with *o*-xylene, blends coated with toluene exhibited stronger tendency toward aggregation and crystalization. This results in more sharper baseline shift observed in blends composed of PM6 and N3 at lower temperature, and more obvious red-shift-then-blue-shift behavior in PM6:Y6 blend.

When shift to stack plot and fit the spectrum with our proposed model, the tendency became more obvious. We found a growing peak centered at 858 nm would suit toluene systems better than 847 nm, wich is one of the extra evidence that toluene facilitates acceptors to stack better than o-xylene. As depicted in the first row of **Supplementary Figure 15**, obvious grow patterns can still be seen in PM6:Y6 blend, and growing portion is seen to be larger for PM6:L8-BO system comapred coating with *o*-xylene. While for PM6:N3 system coated in toluene, the excessive ctystallization nearly ruled out the whole intensity of N3 absorption in dried film. We put the fitting results using our proposed model in second row of **Supplementary Figure 15**. After fitting with our model, the parking position of move peaks seldom changed in dried film in all systems, but the grow peak intensity indeed rose in L8-BO system compared with that coated with *o*-xylene.

Summarizing the findings in the exploration of kinetics in toluene, we find that toluene generally increases the aggregation tendency of acceptors, resulting in higher portion of growing kinetics and heavier crystallization in early stages. Such result is anticipated since toluene molecules face lower resistance when stacking with acceptor backbone compared with *o*-xylene. This could further assist the assembly of acceptors and ease the hindrance brought by side chains. Furthermore, the result proves the generalization ability of our poposed model and aggregation-controlled kinetics.

Supplementary References

1. Virtanen P*, et al.* SciPy 1.0: fundamental algorithms for scientific computing in Python. *Nat Methods* **17**, 261-272 (2020).

2. Harris CR*, et al.* Array programming with NumPy. *Nature* **585**, 357-362 (2020).

3. M. J. Frisch, *et al.* Gaussian 09, Revision D.01. (2016).

4. Zhu L, Zhang J, Guo Y, Yang C, Yi Y, Wei Z. Small Exciton Binding Energies Enabling Direct Charge Photogeneration Towards Low-Driving-Force Organic Solar Cells. *Angew Chem Int Ed Engl* **60**, 15348-15353 (2021).

5. Zhang G*, et al.* Delocalization of exciton and electron wavefunction in non-fullerene acceptor molecules enables efficient organic solar cells. *Nat Commun* **11**, 3943 (2020).

6. Pedregosa F*, et al.* Scikit-learn: Machine Learning in Python. *Journal of Machine Learning Research* **12**, 2825-2830 (2011).
