# Supplementary material for: Concretized structural evolution supported assembly-controlled film-forming kinetics in slot-die coated organic photovoltaics
Source: Nat Commun. 2023 Oct 9;14:6312. doi: 10.1038/s41467-023-42018-7 (PMC10562442; doi:10.1038/s41467-023-42018-7)
Supplement: Supplementary file 3 — Description of Additional Supplementary Files [file 41467_2023_42018_MOESM3_ESM.pdf]

File name: Supplementary Data 1

Description: Fitted raw data of all in-situ UV-vis spectroscopy experiments, spectrum_fitting_raw_results.xlsx.
