# Supplementary material for: Concretized structural evolution supported assembly-controlled film-forming kinetics in slot-die coated organic photovoltaics
Source: Nat Commun. 2023 Oct 9;14:6312. doi: 10.1038/s41467-023-42018-7 (PMC10562442; doi:10.1038/s41467-023-42018-7)
Supplement: Supplementary file 5 — Reporting Summary [file 41467_2023_42018_MOESM5_ESM.pdf]

nature research

Corresponding author(s): Prof. Zhixiang Wei(weizx@nanoctr.cn)

Solar Cells Reporting Summary

Nature Research wishes to improve the reproducibility of the work that we publish. This form is intended for publication with all accepted papers reporting the characterization of photovoltaic devices and provides structure for consistency and transparency in reporting. Some list items might not apply to an individual manuscript, but all fields must be completed for clarity.

For further information on Nature Research policies, including our data availability policy, see Authors & Referees.

Experimental design

Please check: are the following details reported in the manuscript?

1. Dimensions

Item	Yes / No	Response
Area of the tested solar cells	☒ Yes ☐ No	1.036 cm2
Method used to determine the device area	☒ Yes ☐ No	A mask with calibrated area of 1.036cm2 was used to determine the device area.

2. Current-voltage characterization

Item	Yes / No	Response
Current density-voltage (J-V) plots in both forward and backward direction	☐ Yes ☒ No	Hysteresis has not been observed in the organic solar cells (OSCs). J-V plot of best performance cells in reverse direction is provided in supplementary figure S13.
Voltage scan conditions<br>*For instance: scan direction, speed, dwell times*	☒ Yes ☐ No	The scanning step was 0.01 V and dwell time was 30 ms.
Test environment<br>*For instance: characterization temperature, in air or in glove box*	☒ Yes ☐ No	The OSCs were tested in air. The average temperature and relative humidity were 25° C and 30%, respectively.
Protocol for preconditioning of the device before its characterization	☐ Yes ☒ No	No preconditioning was applied prior to device characterization.
Stability of the J-V characteristic<br>*Verified with time evolution of the maximum power point or with the photocurrent at maximum power point; see ref. 7 for details.*	☐ Yes ☒ No	Since this work is not focused on the device stability on continuous light exposure, the evolution of efficiency or current density at maximum power point were not investigated in the manuscript.

3. Hysteresis or any other unusual behaviour

Item	Yes / No	Response
Description of the unusual behaviour observed during the characterization	☐ Yes ☒ No	No hysteresis observed during the characterization in the manuscript.
Related experimental data	☐ Yes ☒ No	No experimental data related.

4. Efficiency

Item	Yes / No	Response
External quantum efficiency (EQE) or incident photons to current efficiency (IPCE)	☒ Yes ☐ No	EQE spectra of best-performing PM6:L8-BO devices were put in supplementary Figure S13.
A comparison between the integrated response under the standard reference spectrum and the response measure under the simulator	☒ Yes ☐ No	The integrated JSC calculated from the EQE curves are comparable to those obtained from the J-V curves.
For tandem solar cells, the bias illumination and bias voltage used for each subcell	☐ Yes ☒ No	No tandem cells were investigated in the manuscript.

5. Calibration

Item	Yes / No	Response
Light source and reference cell or sensor used for the characterization	☒ Yes ☐ No	The J–V measurement was tested via a Keithley 2400 sourcemeter. A solar simulator (SAN-EI ELECTRIC XES-1004SE-200S) with AM 1.5G spectra at 100 mW/cm². The light intensity was calibrated by the certified standard silicon solar cell (Newport Oriel 91150V-KG5).

Confirmation that the reference cell was calibrated and certified

☒ Yes ☐ No

The reference cell with KG5 filter was purchased from Newport Co., Ltd. and was calibrated and certified.

Calculation of spectral mismatch between the reference cell and the devices under test

☒ Yes ☐ No

Spectral mismatch between reference cell and testing cells was less than 5%.

6. Mask/aperture

Size of the mask/aperture used during testing

☒ Yes ☐ No

Size of mask used during the testing is 1.036cm2

Variation of the measured short-circuit current density with the mask/aperture area

☐ Yes ☒ No

Since this work is mainly focused on structural characterization and discussion of kinetics, we did not test our fabricated cells under various mask area.

7. Performance certification

Identity of the independent certification laboratory that confirmed the photovoltaic performance

☐ Yes ☒ No

Since this work is mainly focused on structural characterization and discussion of kinetics, we did not certificate our measured photovoltaic performance.

A copy of any certificate(s)
Provide in Supplementary Information

☐ Yes ☒ No

Since this work is mainly focused on structural characterization and discussion of kinetics, we did not certificate our measured photovoltaic performance.

8. Statistics

Number of solar cells tested

☒ Yes ☐ No

At least 5 independent solar cells fabricated under the same conditions were tested.

Statistical analysis of the device performance

☒ Yes ☐ No

Statistical results of device performance can be found in supplementary table 1.

9. Long-term stability analysis

Type of analysis, bias conditions and environmental conditions
For instance: illumination type, temperature, atmosphere humidity, encapsulation method, preconditioning temperature

☐ Yes ☒ No

Since this work is not focused on the long-term stability, we did not perform long-term stability analysis in the manuscript.
